# Supplementary material for: edgeRun: an R package for sensitive, functionally relevant differential expression discovery using an unconditional exact test
Source: Bioinformatics. 2015 Apr 21;31(15):2589–90. doi: 10.1093/bioinformatics/btv209 (PMC4514933; doi:10.1093/bioinformatics/btv209)
Supplement: Supplementary Data [file supp_31_15_2589__index.html]

edgeRun: an R package for sensitive, functionally relevant differential expression discovery using an unconditional exact test — edgeRun: an R package for sensitive, functionally relevant differential expression discovery using an unconditional exact test — edgeRun: an R package for sensitive, functionally relevant differential expression discovery using an unconditional exact test — Supplementary Data 

# edgeRun: an R package for sensitive, functionally relevant differential expression discovery using an unconditional exact test

## Supplementary Data

files

**Files in this Data Supplement:**

- Supplementary Data - pdf file
