# Supplementary material for: edgeRun: an R package for sensitive, functionally relevant differential expression discovery using an unconditional exact test
Source: Bioinformatics. 2015 Apr 21;31(15):2589–90. doi: 10.1093/bioinformatics/btv209 (PMC4514933; doi:10.1093/bioinformatics/btv209)
Supplement: Supplementary Data [file supp_btv209_supplementary_info.pdf]

`edgeRun`: Supplementary Methods

Emmanuel Dimont*[1], Jiantao Shi[1], Rory Kirchner[1], and Winston Hide[1,2,3]

[1]Department of Biostatistics, Harvard School of Public Health, 655 Huntington Ave, Boston, MA 02115, USA
[2]Harvard Stem Cell Institute, 1350 Massachusetts Ave, Cambridge, MA 02138, USA
[3]Sheffield Institute of Translational Neuroscience, University of Sheffield, 385a Glossop Road, Sheffield, S10 2HQ, United Kingdom

Contents

1 Data Setup **2**

2 Distribution Assumptions **2**

3 Hypothesis of Interest **3**

4 edgeR: Conditional Exact Test (CET) **4**

5 edgeRun: Unconditional Exact Test (UET) **5**

6 Numerical Implementation of the UET **6**
- 6.1 Approximating the Infinite Sum 6
- 6.2 Approximating the Supremum 8

7 Simulation Studies using compcodeR **11**

8 A Real-Data Example **13**
- 8.1 edgeRun v.s. edgeR 13
- 8.2 edgeRun v.s. DESeq2 15

9 Assessing Functional Relevance **16**
- 9.1 Comparison with limma-voom 16

*edimont@mail.harvard.edu

10 References **18**

1 Data Setup

One simple experimental setup in computational biology is one in which the gene expression from 2 different biological conditions (X and Y) is to be compared to one another. We assume that we have n_1 and n_2 replicates of each condition respectively. Next-generation sequencing technologies generate reads or tags that are mapped to a reference genome. The number of tags that map to various genomic loci of interest (e.g. genes) is a measure of that feature's expression. We represent this data in a table in which rows correspond to different genomic loci (e.g. genes), and columns correspond to the biological samples.

	Condition X				Condition Y			
Gene 1	x_{11}	x_{12}	$\cdots$	x_{1n_1}	y_{11}	y_{12}	$\cdots$	y_{1n_2}
Gene 2	x_{21}	x_{22}	$\cdots$	x_{2n_1}	y_{21}	y_{22}	$\cdots$	y_{2n_2}
$\vdots$	$\vdots$	$\vdots$	$\ddots$	$\vdots$	$\vdots$	$\vdots$	$\ddots$	$\vdots$
Gene G	x_{G1}	x_{G2}	$\cdots$	x_{Gn_1}	y_{G1}	y_{G2}	$\cdots$	y_{Gn_2}

2 Distribution Assumptions

The simplest model for an integer count variable X that is not assumed to be bounded above is the Poisson distribution that has the following probability mass function (p.m.f.):

$$X \sim \text{Poisson}(\mu)$$

$$P(X = x|\mu) = \frac{\mu^x e^{-\mu}}{x!}$$

where $\mathrm{E}[X] = \mu$ and $\mathrm{Var}[X] = \mu$. One major problem with the Poisson model is the strong assumption that the variance is equal to the mean. In practice this assumption rarely holds. To obtain a model that allows for more variation than that assumed by the Poisson, we can assume that the Poisson mean parameter has a distribution of its own rather than being a fixed constant. The simplest distribution for a non-negative continuous mean parameter is the Gamma distribution with the probability density function (p.d.f.) given below. We now have have the hierarchical model:

$$X|M \sim \text{Poisson}(M) \text{ and } M \sim \text{Gamma}(\alpha, \beta)$$

$$f_M(m|\alpha, \beta) = \frac{1}{\Gamma(\alpha)\beta^\alpha} m^{\alpha-1} e^{-\frac{1}{\beta}m}$$

This is also called Gamma *mixing*. Here, M is the random variable and m is the realization of the Poisson mean parameter. For the Gamma distribution, α is the shape and β is the scale parameter respectively. As a consequence,

$\mathrm{E}[M] = \alpha\beta$ and $\mathrm{Var}[M] = \alpha\beta^2$. If we let $\alpha = 1/\phi$ and $\beta = \phi\mu$, we then have $\mathrm{E}[M] = \mu$ and $\mathrm{Var}[M] = \phi\mu^2$. With repeated sampling on average, the mean parameter will be μ, but it will have some variation that depends on a new parameter ϕ which we call the *dispersion.* The marginal distribution of X under this structure results in the *negative binomial* distribution with p.m.f.:

$$X \sim \mathrm{NegBin}(\mu, \phi)$$

$$P(X = x|\mu, \phi) = \binom{x + \phi^{-1} - 1}{x} \left(\frac{1}{1 + \phi\mu}\right)^{\phi^{-1}} \left(\frac{\phi\mu}{1 + \phi\mu}\right)^{x}$$

$$\mathrm{E}[X] = \mu \text{ and } \mathrm{Var}[X] = \mu + \phi\mu^2$$

Using this model, X allows for extra-Poisson variation which we call *overdispersion*, using the parameter ϕ.

With this distribution in place, we now make the following assumptions concerning our data:

$$X_{gr} \overset{iid}{\sim} \mathrm{NegBin}(\mu_g, \phi_g)$$

$$Y_{gr} \overset{iid}{\sim} \mathrm{NegBin}(\mu'_g, \phi_g)$$

Each gene is allowed to have a separate mean and/or dispersion, and X and Y can have different means as well. Independence of samples is assumed.

Note: In practice, each sample will have a different number of total tags that are generated and successfully mapped, i.e. the *sequencing depth* or *library size.*

$$X_{gr} \overset{iid}{\sim} \mathrm{NegBin}(k_r\lambda_g, \phi_g)$$

As a result, μ is equal to the product of k, the library size of the sample and λ, the relative expression of the gene. To make all of the samples comparable requires a procedure called *normalization.* It is beyond the scope of this paper to discuss *normalization* methods, and the choice of any one method does not affect our discussion that follows. We proceed by assuming that X and Y are transformed into *pseudo-counts* in all further analyses as is standard procedure. For this reason and for clarity, we revert back to our original notation.

3 Hypothesis of Interest

We are interested in testing the G hypotheses of the form:

$$H_0 : \mu_g = \mu'_g$$

$$H_0 : \mu_g \neq \mu'_g$$

Since sample sizes are typically small when sequencing technologies are expensive, we proceed with an *exact* test that makes no asymptotic assumptions.

The *p-value* is defined as the probability of getting something as or more extreme as the observed data under the null hypothesis. Obtaining the probability of the observed data is straightforward:

$$P(\text{observed}_g) = P(X_{g1} = x_{g1} \cap \cdots \cap X_{gn_1} = x_{gn_1} \cap Y_{g1} = y_{g1} \cap \cdots \cap Y_{gn_2} = y_{gn_2})$$

The challenge is to identify which data points are as or more extreme than those observed. The trivial approach would require the enumeration of $n_1 n_2$ variables, but this is not feasible. For a particular gene, let $S_{g1} = \sum X_g$ and $S_{g2} = \sum Y_g$. It becomes much easier to work with the probabilities associated with these sums rather than the original random variables. The sum of iid negative binomial random variables is also negative binomial:

$$S_{g1} \sim \text{NegBin}(n_1\mu_g, \phi_g/n_1)$$

$$S_{g2} \sim \text{NegBin}(n_2\mu_g, \phi_g/n_2)$$

We can now calculate the probabilities of various (S_1, S_2) independent pairs as follows:

$$P(s_{g1}, s_{g2}|\mu_g) = P(S_{g1} = s_{g1} \cap S_{g2} = s_{g2}|\mu_g) =$$

$$\binom{s_{g1} + n_1/\phi_g - 1}{s_{g1}} \binom{s_{g2} + n_2/\phi_g - 1}{s_{g2}} \left(\frac{1}{1+\phi_g\mu_g}\right)^{\frac{n_1+n_2}{\phi_g}} \left(\frac{\phi_g\mu_g}{1+\phi_g\mu_g}\right)^{s_{g1}+s_{g2}}$$

We can see that any p-value obtained from this formula will depend on the choice of ϕ_g and μ_g. However, our hypothesis of interest does not depend on these parameters. Under the null, we only assume that the two means are equal to one another and we make no statements about what that value is. As a result, both ϕ_g and μ_g are *nuisance* parameters that need to be eliminated. There is no generally accepted way to eliminate ϕ_g, and so in all ensuing discussion, ϕ_g is assumed to be a known constant. On the other hand there are two ways to perform the elimination of μ_g, one resulting in a *conditional* exact test (CET) and the other, an *unconditional* exact test (UET).

4 edgeR: Conditional Exact Test (CET)

The popular `edgeR` Bioconductor package (Robinson et al., 2010) implements an exact test that eliminates the nuisance mean parameter by conditioning on a sufficient statistic for the mean. This technique was first proposed by Fisher (1925) to eliminate the nuisance parameter when testing equality of parameters in the *binomial* distribution, resulting in Fisher's Exact Test. The sufficient statistic is the total sum $S_g = S_{g1} + S_{g2}$. The p-value is calculated based on the following conditional probability. **Reminder:** The parameter ϕ_g, whether it is estimated from the data or not, is assumed to be known. For more details on how the dispersion is estimated, see Robinson and Smyth (2007) and Robinson et al. (2010).

$$P(S_{g1} = s_{g1} \cap S_{g2} = s_{g2}|S_g) = \frac{P(S_{g1} \cap S_{g2} \cap S_g)}{P(S_g)} = \frac{P(S_{g1} = s_{g1} \cap S_{g2} = s_g - s_{g1})}{P(S_g)}$$

$$= \frac{\binom{s_{g1}+(n_1/\phi_g)-1}{s_{g1}}\binom{s_g-s_{g1}+(n_2/\phi_g)-1}{s_g-s_{g1}}}{\binom{s_g+((n_1+n_2)/\phi_g)-1}{s_g}} = CP(s_{g1})$$

We can see that this expression no longer depends on μ. S_{g1} is taken as a statistic to determine values which are as or more extreme as those observed. The *conditional* two-sided p-value can then be calculated as follows:

$$p_{\texttt{edgeR}} = 2 \times \min\left\{\sum_{k=0}^{\widehat{s_{g1}}} CP(k), \sum_{k=\widehat{s_{g1}}}^{\widehat{s_g}} CP(k)\right\}$$

This is the default method used in the `exactTest` function in `edgeR`. It is called the *double-tail* method because it calculates both tails of the conditional probability and doubles the smallest of the two.

The p-value from the conditional exact test is very easy to compute, however this approach suffers from a loss in power due to the fact that conditioning is performed. The smaller the value of S_g (e.g. few replicates are available and/or the gene has low expression levels), the greater the loss in power. This happens because a conditional probability by definition restricts the original sample space to a smaller subset. If S_g is small, then $CP(s_{g1})$ may have a small finite number of values that it can take, but since it must sum to 1, the values are all larger, leading to a larger p-value. For a more in-depth discussion of why this loss of power occurs, see Mehta and Senchaudhuri (2003) and Casella and Berger (2002, p.399).

5 edgeRun: Unconditional Exact Test (UET)

We can eliminate the loss in power due to conditioning by performing an alternative exact test that eliminates the nuisance mean parameter in a different way. Instead of working with the conditional probability $CP(s_{g1})$, we use the unconditional probability of the observed data $P(s_{g1}, s_{g2})$ as a basis for calculating a p-value. One challenge involves determining what set of data points s_{g1} and s_{g2} correspond to values as or more extreme than those observed. We use the *pooled* z-statistic T for this purpose:

$$T(s_{g1}, s_{g2}) = \frac{s_{g1} - s_{g2}}{\sqrt{\left(\bar{\mu}_g + \phi_g \bar{\mu}_g^2\right)(n_1 + n_2)}} \text{ where } \bar{\mu}_g = \frac{s_{g1} + s_{g2}}{n_1 + n_2}$$

This statistic is adapted from the z-statistic used for a *z-test* for testing the equality of two normal means assuming equal variances with some minor modifications. The numerator is simply the difference in the sums of the two groups, while the denominator is the standard error of this difference, assuming independent negative binomially distributed data with an estimated common mean $\bar{\mu}_g$. The observed sums yield the value $T_0 = T(\widehat{s_{g1}}, \widehat{s_{g2}})$. Larger values of the

statistic $|T|$ relative to $|T_0|$ (or alternatively T^2 relative to T_0^2) correspond to increasing evidence of deviation from what we get using the observed data.

We can calculate T for every combination of (s_{g1}, s_{g2}) and compare it with T_0 to determine if that combination is as or more extreme as the one observed. For those values of (s_{g1}, s_{g2}) that are as or more extreme as those observed, we calculate their unconditional probability P, and then sum these probabilities across all such points. This process is then repeated for different values of μ_g until a supremum is obtained, i.e. take the largest sum of P as the two-sided p-value:

$$p_{\texttt{edgeRun}} = \sup_{\mu_g} \left\{ \sum_{\{(j,k):T(j,k)^2 \geq T_0^2\}} P(j,k|\mu_g) \right\}$$

Reminder: Just like in the CET, the parameter ϕ_g present in P, whether it is estimated from the data or not, is assumed to be known.

This approach of eliminating the nuisance parameter by maximizing over it can be traced back to Barnard (1945) in which he proposed a similar test for binomially distributed data as an alternative to Fisher's exact test. Barnard showed that this technique yields a test that is more powerful than Fisher's.

The main disadvantage of this approach however is the difficulty in its implementation. Unlike in the conditional p-value calculation, the number of terms to be summed in this expression is infinite. This, together with the fact that there is no closed-form solution to the supremum operator, requires the use of numerical techniques to obtain this p-value which is much more computationally intensive.

6 Numerical Implementation of the UET

6.1 Approximating the Infinite Sum

Ignoring the supremum operation for now, one major challenge in implementing the UET is performing the summation of probabilities across the relevant space of s_{g1} and s_{g2}. One could propose to calculate values of T for all such points, determine which points are relevant, and then perform the summation over these points. This is impractical since the space over which this needs to be done is the upper right quadrant of integers which is countably infinite. The space in question is depicted in the figure below. The red diagonal line corresponds to the null case where $s_{g1} = s_{g2}$. Points in red are those for which $T^2 < T_0^2$ and so belong to the null as well. Points in black are those for which $T^2 \geq T_0^2$ and are those over which we want to perform the summation. Let A_0 be the total probability mass occupied by the null points depicted in red and A_1 its complement. The probability mass of black points is then $A_1 = 1 - A_0$, and the problem is simplified if we can find a way to characterize the red area. This summation still requires summing over an infinite array over 2 dimensions. We

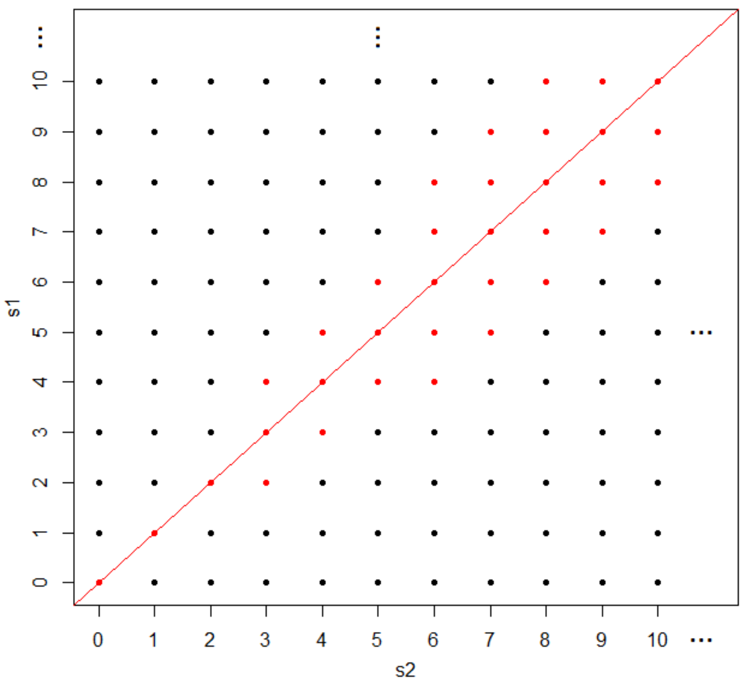

Figure 1: The Cartesian space of (s_{g1}, s_{g2}). Red points: $T < T_0$

can reduce this to an infinite summation over just 1 dimension as follows:

$$A_1 = \sum_{\left\{(j,k):T(j,k)^2 \geq T_0^2\right\}} P(j,k|\mu_g)$$

$$= \lim_{K\to\infty} \sum_{s_{g2}=0}^{K} \left\{1 - \left[F(s_{g1}^{U|s_{g2}}) - F(s_{g1}^{L-1|s_{g2}})\right]\right\} P(S_{g2} = s_{g2})$$

In the above expression, $P(S_{g2})$ is the marginal p.m.f. of S_{g2} with mean $n_2\mu_g$ and dispersion ϕ_g/n_2 and $F(\cdot)$ is the c.d.f. of S_{g1} with mean $n_1\mu_g$ and dispersion ϕ_g/n_1. s_{g1}^U and s_{g1}^L correspond to the values of s_{g1} which are the edge points on vertical slices of the red probability mass area for a given value of s_{g2}. In essence what the expression above is doing is calculating the complementary mass of the red area (i.e. the black area) by taking vertical slices across values of s_{g2}.

The next challenge is finding these edge points that are used in the c.d.f. which we find by solving the inequality $T^2 < T_0^2$.

$$\text{Let:} \quad w_1 = 1 + \frac{\phi_g T_0^2}{n_1 + n_2} \quad \text{and} \quad w_2 = 1 - \frac{\phi_g T_0^2}{n_1 + n_2}$$

After some algebraic manipulation, we get the following quadratic inequality:

$$as_{g1}^2 + bs_{g1} + c < 0 \quad \text{where:}$$

$$a = w_2 \quad b = -(2w_1 s_{g2} + T_0^2) \quad c = s_{g2}(w_2 s_{g2} - T_0^2)$$

The solutions are obtained by applying the quadratic formula. Let L be the smallest and U be the largest of the two solutions. Because $w_2 > 0$ always, the parabola is convex, and since the solutions must be integers, we apply the appropriate floor and ceiling operators.

$$s_{g1}^{L|s_{g2}} = \max(\lceil L \rceil, 0)$$

$$s_{g1}^{U|s_{g2}} = \max(\lfloor U \rfloor, 0)$$

Finally, we take the maximum of the solution with 0 to avoid solutions which are negative. It should be noted that these solutions depend on the specific value of s_{g2}.

We approximate the infinite sum in A_1 by choosing an upper bound K which is sufficiently large. Larger values of K increase computing time but increase the accuracy of the p-value. The figure below shows how the level of accuracy of the p-value increases with K. Two groups with 3, 5 and 10 samples per group are simulated under the null with $s_{g1} = s_{g2} = 10,000$ for various levels of ϕ. Since this is a null scenario, we expect a true p-value of 1, which as expected, is evident as the asymptote for every curve in the plot. In addition to the p-value, the y-axis in this figure also refers to the goodness of the approximation. Larger values of K are necessary for a good p-value approximation for increasing ϕ and decreasing sample size with fixed s_g (i.e. increasing observed average tag counts per replicate).

By default, `edgeRun` takes $K = 50,000$ as a compromise between accuracy and speed, but this can be adjusted by the user. As seen from the figure, this value of K yields approximately 80% accuracy for a wide range of data scenarios. On an Intel® Core-i7 4700HQ 2.4GHz processor, a computation with 20,000 genes takes approximately 15 minutes to complete. For a faster analysis, we suggest starting with `edgeR` which can generate differential expression results in less than a minute. **NB:** The default value of K could be inadequate in some situations (highly expressed genes or large sample size) and could lead to some loss in FDR control (the true false discovery rate will be higher than expected). In such cases, we recommend increasing K to 100,000 or higher.

6.2 Approximating the Supremum

In the previous section, we find an approximation to A_1. The next challenge is to find the value of μ_g that will maximize the value of A_1 which we call μ^*, i.e.

$$\mu_g^* = \arg\max_{\mu_g} A_1$$

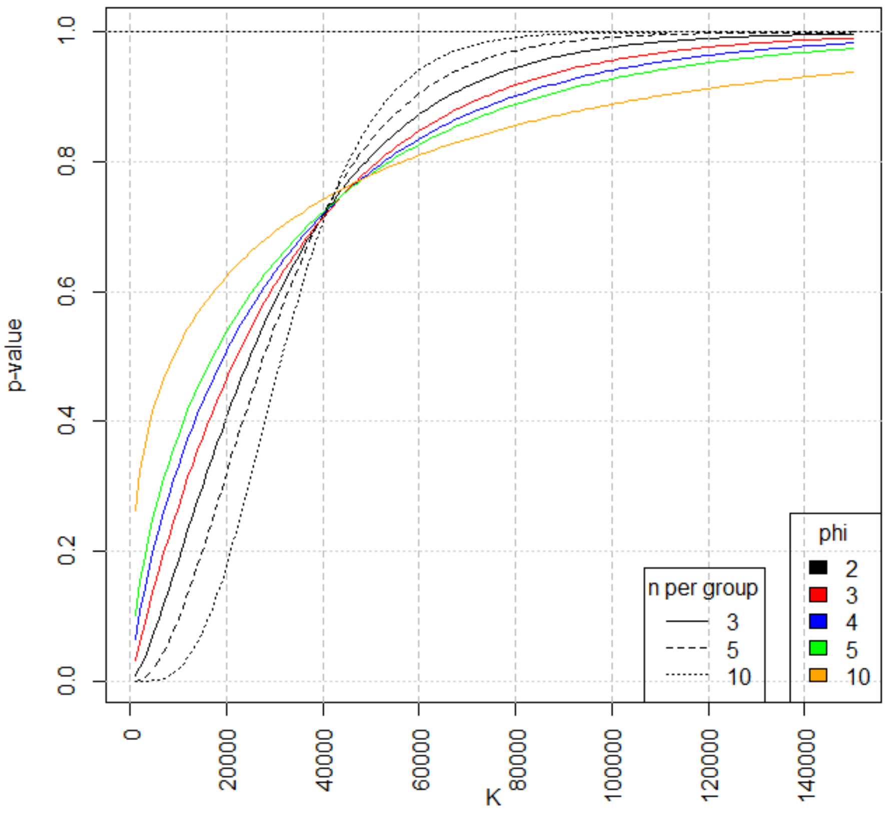

Figure 2: Relationship between p-value accuracy and K for various levels of ϕ and number of replicates per group with $s_{g1} = s_{g2} = 10,000$

To solve this problem, we attempt to find a relationship between the μ^* and various factors. To do this, data was simulated under the non-null case for arbitrarily chosen $s_{g1} = 1000$ and $s_{g2} = 1$ and $n_1 = n_2 = 2$ across a range of ϕ_g which were chosen over a range 0-20 in fine increments of 0.01. For each case, μ^* was obtained by evaluating A_1 over an iterative logarithmic grid of μ_g values until an accuracy within ± 1 was obtained. These values are plotted in the figure below.

This relationship is characterized and stored in `edgeRun` as follows:

$$\mu^*(\phi_g) = \begin{cases} 22.98 & : \quad 0 \leq \phi_g < 0.20 \\ -74,939 + 374,809\phi_g & : \quad 0.20 \leq \phi_g < 0.32 \\ \sum_{i=0}^{11} \beta_i \phi_g^i & : \quad 0.32 \leq \phi_g < 20 \\ \sum_{i=0}^{11} \beta_i 20^i & : \quad 20 \leq \phi_g \end{cases}$$

Very small values of $\phi_g < 0.2$ were simulated using the Poisson distribution since the negative binomial routines were found to be unstable in this range. A

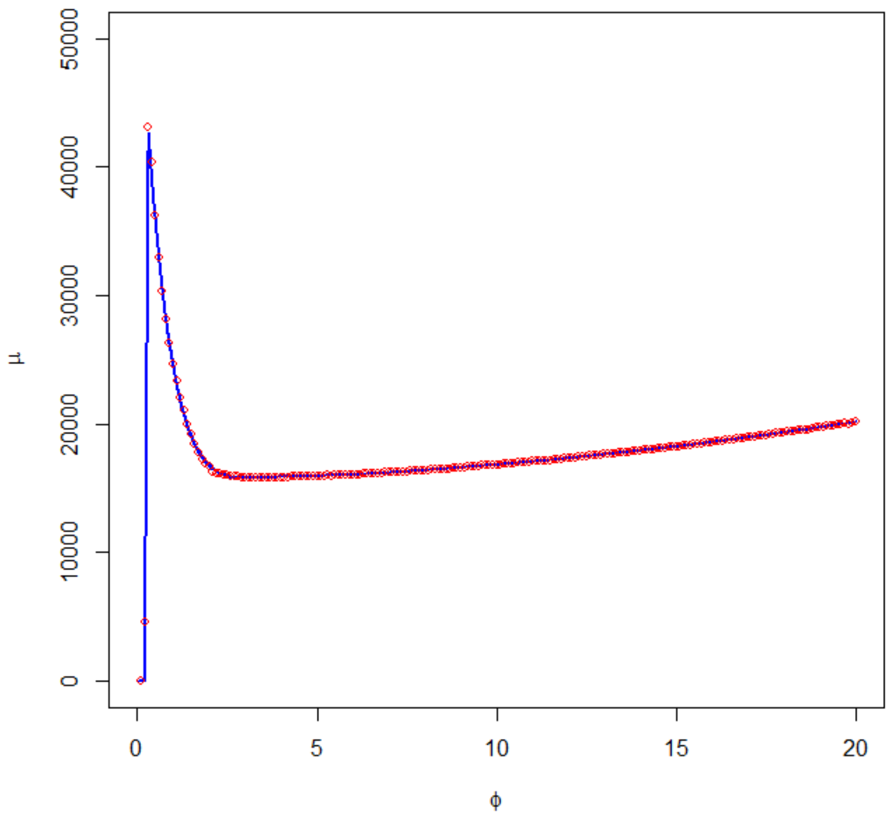

Figure 3: Relationship between μ^* (μ_g that maximizes A_1), v.s. ϕ_g for the case $s_{g1} = 1000, s_{g2} = 1$ and $n_1 = n_2 = 2$. Red points correspond to estimated maximum values using an iterative logarithmic grid. Blue line is the fitted approximation.

discontinuity was detected in the from 0.2-0.32 and it was very difficult to obtain a result in this range, hence a linear interpolation was performed to join to the upper range of ϕ_g. An 11th order polynomial fit was found to approximate the solution for $\phi_g > 0.32$ very well with an R^2 very close to 100%. The β coefficients of the polynomial approximation are stored in the `fit.2` object. For the remaining range of $\phi_g > 20$, since the curve appears to level off, the accuracy of using the exact value of ϕ_g v.s. the upper threshold of 20 in the polynomial fit was not significantly altered (data no shown).

It was found that varying the values of n_1 and n_2 affected this relationship

only by the corresponding scaling effect on μ_g and ϕ_g. As a result, the case of $n_1 = n_2 = 2$ is taken as a reference and all data for other n is linearly scaled to the $n = 2$ case for the purposes of obtaining μ^*. Finally, values of s_{g1} and s_{g2} were chosen to lie in the non-null space, but the numbers were chosen arbitrarily. We choose values in the non-null case because we want to be conservative specifically for data that is non-null, since values that are closer to the null are more likely to be non-significant anyway. We find that the solution for μ^* does not significantly alter if other values for s are used (data not shown). Once again, this approximation is a compromise between accuracy and speed.

7 Simulation Studies using compcodeR

We used the Bioconductor package `compcodeR` (Soneson, C., 2014) to benchmark the performance of `edgeRun` against a panel of 26 other differential expression tools using various parameters. We used the B_{625}^{625} simulated dataset with 2 replicates per condition in which 10% of a total of 12500 genes were differentially expressed (625 genes in each condition). More details on how these random testing datasets are generated can be found in Soneson and Delorenzi (2013). The figures below show the performance of `edgeRun` in terms of area under the curve (AUC) and the true positive rate (TPR). The blue line corresponds to the value attained by `edgeRun`.

We find that `edgeRun` has the highest AUC of all methods tested, meaning that on the average, choosing across a range of cutoff values of the false discovery rate (FDR) to determine which genes to call as differentially expressed (e.g. call a gene differentially expressed if it's adjusted p-value < 0.05), `edgeRun` has the most optimal combination of sensitivity (true positive rate) and specificity (true negative rate).

Typically however, instead of looking across a range of cutoff values, in practice a single cutoff value is used to determine differentially expressed genes, one of the most popular being the 5% cutoff for adjusted p-values. In the next figure we can see that the sensitivity or true positive rate is high, but no longer the best across all tools compared. We note that `DESeq2` (Love, M.I. et al. 2014) is by far the most competitive, with sensitivities reaching nearly 3 times that of `edgeRun`. There is always a trade-off between sensitivity and specificity however, and in the figure below we can see how the false discovery rate is higher for those `DESeq2` settings that yielded the highest TPR.

We concede that `DESeq2` can be more powerful than `edgeRun`, however in the next several sections we propose a new approach of comparing these tools from a functional relevance perspective. As a result, from now on we only focus on the comparison between `edgeRun` and `DESeq2`.

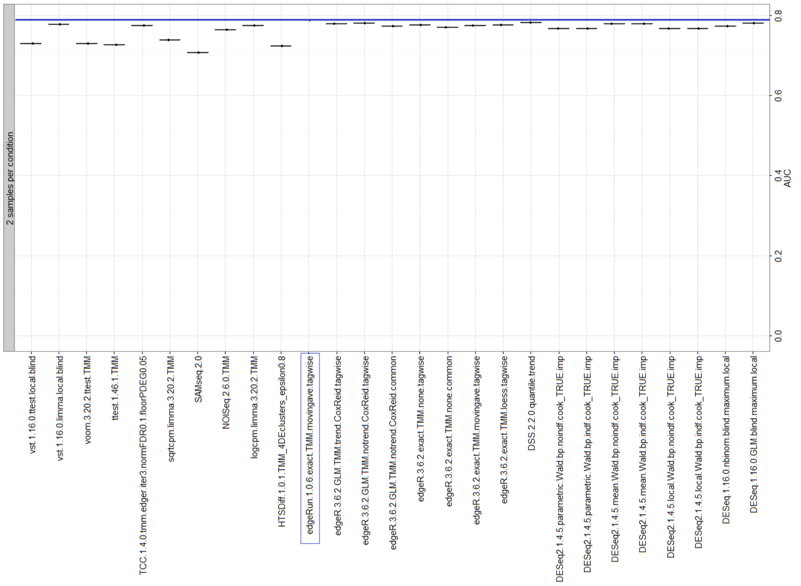

Figure 4: Area under the curve (AUC) comparison between `edgeRun` (blue) and 26 other tools/parameter combinations using B_{625}^{625} simulation dataset

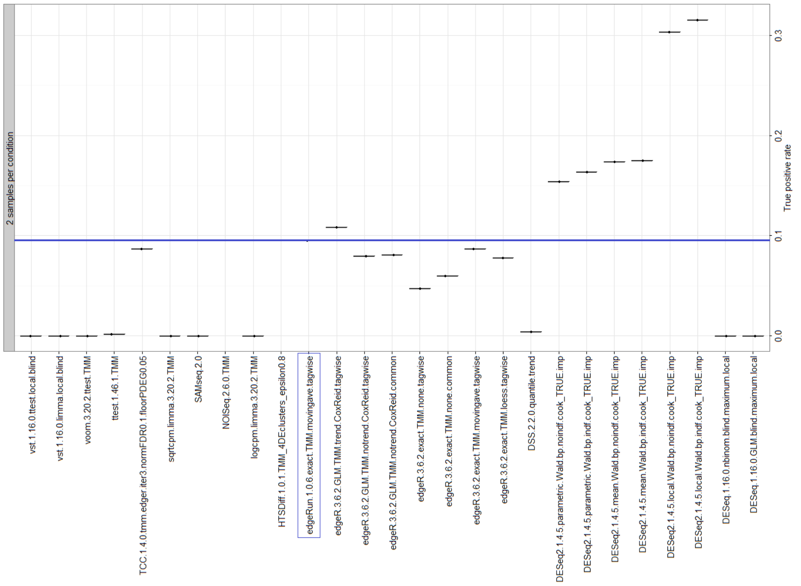

Figure 5: True positive rate (TPR) comparison at 5% cutoff between `edgeRun` (blue) and 26 other tools/parameter combinations using B_{625}^{625} simulation dataset

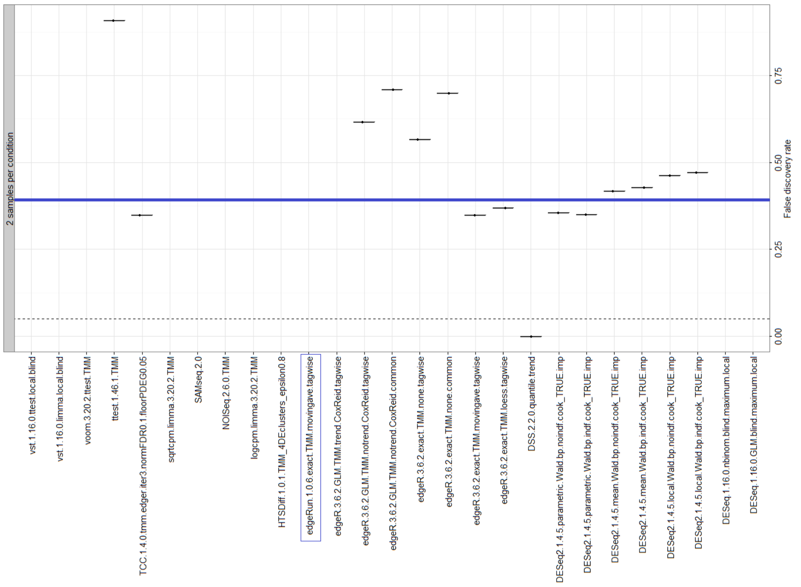

Figure 6: False discovery rate (FDR) comparison at 5% cutoff between `edgeRun` (blue) and 26 other tools/parameter combinations using B_{625}^{625} simulation dataset

8 A Real-Data Example

Having shown that `edgeRun` performs well on simulated data, we next proceed to applying it to some real experimental data. For this purpose we use the prostate cancer model by Li et al. (2008), the same dataset used by the authors of `edgeR` (Robinson et al. 2010) to demonstrate its functionality. The dataset consists of a two sample comparison with $n_1 = 3$ and $n_2 = 4$.

8.1 edgeRun v.s. edgeR

We first compare `edgeRun` to `edgeR`. We can see in the MA plot below that there is wide overlap in the genes called significant at a 5% FDR cutoff between the two methods. We can also see that `edgeRun` is more sensitive than `edgeR` since it is able to call genes significant that for a fixed level of expression, have lower fold change. We see this increased power across the whole range of expression levels, but especially so for more lowly expressed genes as expected. It should be noted that `edgeRun` calls as significant all those genes already called by `edgeR`, thus confirming in practice the expected theoretical gains in power due to the UET over the CET.

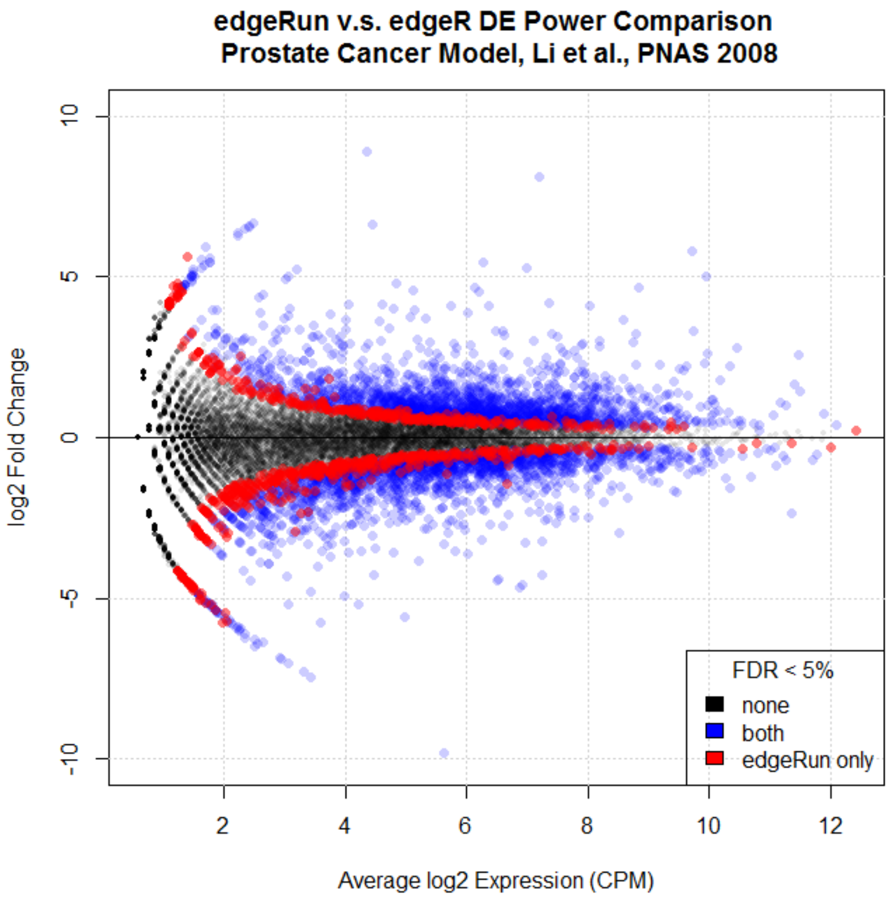

Figure 7: `edgeRun` (UET) is uniformly more powerful than `edgeR` (CET)

8.2 edgeRun v.s. DESeq2

Previously we have shown in simulations that `DESeq2` is the only other tool that was shown to be more powerful than `edgeRun`. We now perform a comparison between the `edgeR` family (`edgeRun` and `edgeR`) and the `DESeq` family (`DESeq` and `DESeq2`) on the same dataset, the results of which can be seen in the MA plot below. Once again we can see that there is wide agreement between all 4 tools, however we find interesting differences in what classes of genes the different families call as significant. The `DESeq` family is more sensitive at detecting genes with higher expression levels, whereas `edgeRun` is more sensitive at the lower end of the gene expression spectrum.

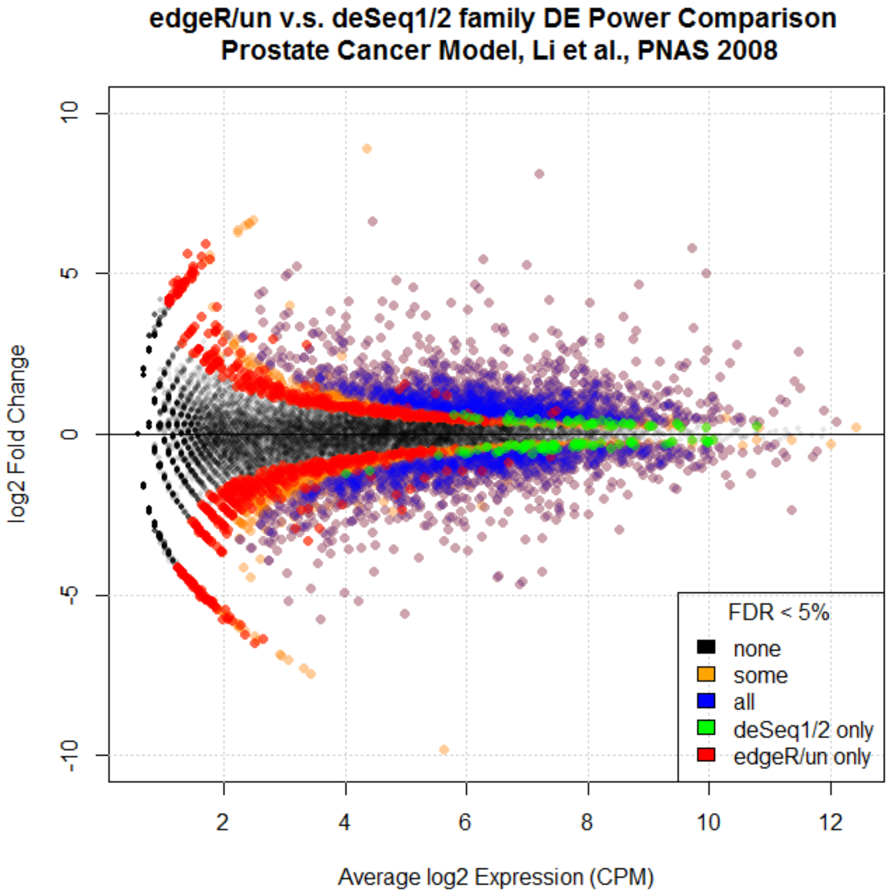

Figure 8: `edgeRun` is more sensitive for lowly, whereas `DESeq2` is more sensitive for more highly expressed genes

9 Assessing Functional Relevance

As described in the main manuscript, Out of the 4226 genes reported as differentially expressed in a prostate cancer dataset, 80% were common to both `edgeRun` and `DESeq2`. We define these shared genes as consensus genes, which are assumed to be truly differentially expressed (DE). Although `edgeRun` identified 6 times more DE genes compared to `DESeq2` (740 vs. 112), we cannot simply say all the called genes are true DE genes. But its reasonable to hypothesize that true DE genes are functionally related to consensus genes. We thus used GRAIL (Raychaudhuri et al., 2009) coupled with a global coexpression networks COXPRESdb (Obayashi et al., 2013) to assess the significance of functional relatedness between a gene and the consensus group. GRAIL builds coexpression subnetworks using provided seed genes, and then assesses the relatedness between a query gene and seed networks. To avoid the heterogeneity of subnetworks, we split the consensus genes into up-regulated and down-regulated groups, and only select top 500 genes (by fold change) for each group. Using a cutoff of false discovery rate (FDR) of 5%, more than 40% of genes in each consensus group are significantly correlated to other genes in the same group, suggesting that genes in the consensus group form tightly connected subnetworks. By checking the genes uniquely called by two tools, we can see the genes reported by `edgeRun` are more likely to be functionally relevant (14.7% vs. 10.7% with Up seeds, and 18.5% vs. 6.2% with Down seeds).

9.1 Comparison with limma-voom

We also performed a comparison of functional relevance of uniquely detected genes between `edgeRun` and `limma-voom`, the results of which can be seen below. Once again, a large group of commonly differentially expressed genes were detected, showing general agreement between the two methods. Again we could see that a large proportion of consensus genes form tightly connected subnetworks. However `edgeRun` found 2.3 times more genes uniquely differentially expressed. Unlike in the previous comparison though, `limma-voom` was found to have a slightly higher proportions of all unique genes that were functionally relevant (37.5 % v.s. 34.4 %). This difference was not found to be statistically significant however (p-value = 0.54). Hence, the functional relevance proportion of both tools is indistinguishable, but `edgeRun` provides more genes for analysis.

A

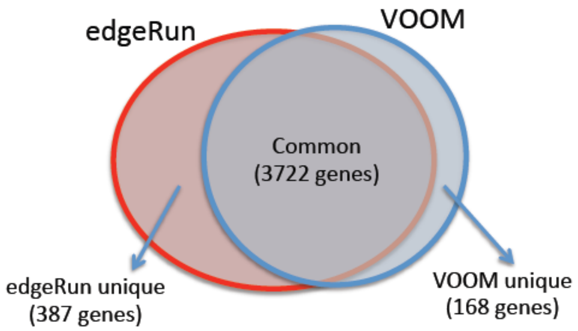

B

	# Genes	# / % genes correlated to UP seeds	# / % genes correlated to DN seeds
Common UP	500	155 / 31.0%	-
Common DN	500	-	213 / 42.6%
edgeRun Unique	387	21 / 5.4%	112 / 28.9%
VOOM Unique	168	13 / 7.7%	50 / 29.8%

Figure 9: `edgeRun` v.s. `limma-voom` in terms of functional relevance

10 References

Barnard, G.A. (1945). A new test for 2x2 tables. *Nature*. 156:177.

Casella, G. and Berger, R.L. (2002). Statistical Inference. Second Edition. Duxbury, Thomas Learning Inc, Pacific Grove, CA.

Fisher, R.A. (1925). Statistical Methods for Research Workers. Oliver and Boyd, Edinburgh.

Li, H., et al. (2008). Determination of tag density required for digital transcriptome analysis: application to androgen-sensitive prostate cancer model. *PNAS* 105(51):20179-84.

Love, M.I., Huber, W., and Anders, S. (2014). Moderated estimation of fold change and dispersion for RNA-Seq data with DESeq2. *bioRxiv* http://dx.doi.org/10.1101/002832

Mehta, C.R. and Senchaudhuri, P. (2003). Conditional vs Unconditional Exact Tests for Comparing Two Binomials. *Cytel Software Corporation.* http://www.cytel.com/papers/twobinomials.pdf

Obayashi,T. et al. (2013) COXPRESdb: a database of comparative gene coexpression networks of eleven species for mammals. Nucleic Acids Res., 41, D101420.

Raychaudhuri,S. et al. (2009) Identifying relationships among genomic disease regions: predicting genes at pathogenic SNP associations and rare deletions. *PLoS Genetics*, 5, e1000534.

Robinson, M.D., and Smyth, G.K. (2007). Small sample estimation of negative binomial dispersion, with applications to SAGE data. *Biostatistics* 9:2, 321-332.

Robinson, M.D., McCarthy, D.J., Smyth, G.K. (2010). edgeR: a Bioconductor package for differential expression analysis of digital gene expression data. *Bioinformatics* 26, 139-140.

Soneson, C. and Delorenzi M. (2013). A comparison of methods for differential expression analysis of RNA-seq data. *BMC Bioinformatics* 14:91.

Soneson, C. (2014). compcodeR: an R package for benchmarking differential expression methods for RNA-seq data. *Bioinformatics* May 9. pii: btu324.
